# Neonatal NET-Inhibitory Factor improves survival in the cecal ligation and puncture model of polymicrobial sepsis by inhibiting neutrophil extracellular traps

**DOI:** 10.3389/fimmu.2022.1046574

**Published:** 2023-01-17

**Authors:** Claudia V. de Araujo, Frederik Denorme, W. Zac Stephens, Qing Li, Mark J. Cody, Jacob L. Crandell, Aaron C. Petrey, Kimberly A. Queisser, John L. Rustad, James M. Fulcher, Judah L. Evangelista, Michael S. Kay, Joshua D. Schiffman, Robert A. Campbell, Christian C. Yost

**Affiliations:** ^1^ Department of Pediatrics/Neonatology, University of Utah, Salt Lake City, UT, United States; ^2^ Molecular Medicine Program, University of Utah, Salt Lake City, UT, United States; ^3^ Department of Pathology, University of Utah, Salt Lake City, UT, United States; ^4^ High Throughput Genomics and Bioinformatic Analysis Shared Resource, Huntsman Cancer Institute, University of Utah, Salt Lake City, UT, United States; ^5^ Department of Biochemistry, University of Utah, Salt Lake City, UT, United States; ^6^ Department of Pediatrics/Hematology-Oncology, University of Utah, Salt Lake City, UT, United States; ^7^ Peel Therapeutics, Inc., Salt Lake City, UT, United States; ^8^ Department of Internal Medicine, University of Utah, Salt Lake City, UT, United States

**Keywords:** sepsis, neutrophil, neutrophil extracellular trap, neonatal NET-Inhibitory Factor, cecal ligation and puncture, microbiome, antibiotic resistance, innate immunity

## Abstract

**Introduction:**

Neutrophil extracellular traps (NETs) clear pathogens but may contribute Q8 pathogenically to host inflammatory tissue damage during sepsis. Innovative therapeutic agents targeting NET formation and their potentially harmful collateral effects remain understudied.

**Methods:**

We investigated a novel therapeutic agent, neonatal NET-Inhibitory Factor (nNIF), in a mouse model of experimental sepsis – cecal ligation and puncture (CLP). We administered 2 doses of nNIF (1 mg/ kg) or its scrambled peptide control intravenously 4 and 10 hours after CLP treatment and assessed survival, peritoneal fluid and plasma NET formation using the MPO-DNA ELISA, aerobic bacterial colony forming units (CFU) using serial dilution and culture, peritoneal fluid and stool microbiomes using 16S rRNA gene sequencing, and inflammatory cytokine levels using a multiplexed cytokine array. Meropenem (25 mg/kg) treatment served as a clinically relevant treatment for infection.

**Results:**

We observed increased 6-day survival rates in nNIF (73%) and meropenem (80%) treated mice compared to controls (0%). nNIF decreased NET formation compared to controls, while meropenem did not impact NET formation. nNIF treatment led to increased peritoneal fluid and plasma bacterial CFUs consistent with loss of NET-mediated extracellular microbial killing, while nNIF treatment alone did not alter the peritoneal fluid and stool microbiomes compared to vehicle-treated CLP mice. nNIF treatment also decreased peritoneal TNF-a inflammatory cytokine levels compared to scrambled peptide control. Furthermore, adjunctive nNIF increased survival in a model of sub-optimal meropenem treatment (90% v 40%) in CLP-treated mice.

**Discussion:**

Thus, our data demonstrate that nNIF inhibits NET formation in a translationally relevant mouse model of sepsis, improves survival when given as monotherapy or as an adjuvant with antibiotics, and may play an important protective role in sepsis.

## Introduction

Sepsis represents a maladaptive immune response to infection that can lead to multiorgan failure with significant morbidity and mortality (1). Best estimates on the global disease burden of sepsis extrapolated from data captured by developed nations suggests that 31.5 million cases of sepsis and 19.4 million cases of severe sepsis occur globally per annum, with potentially 5.3 million deaths due to sepsis each year (2). Sepsis-induced organ dysfunction and mortality are attributed to the interplay between inflammatory and anti-inflammatory host responses (3, 4). These host responses result from the interaction of pathogen-associated molecular patterns (PAMPs) with pattern recognition receptors (PRRs), which mediate the host response to specific pathogens. PRRs may also be activated by host nuclear, mitochondrial, and cytosolic proteins, known as damage-associated molecular patterns (DAMPs), released from injured cells during sepsis (5, 6). The polymorphonuclear leukocyte (PMN; neutrophil) is the most abundant circulating leukocyte and responds first to PAMP- and DAMP-associated molecular triggers released during infection. As an essential component of innate immunity, neutrophils contain and kill pathogens to promote survival despite infection (7, 8). Neutrophils phagocytize and eradicate pathogens intracellularly through oxidative and nonoxidative processes activated simultaneously with phagocytosis. Although destruction of infectious agents often occurs intracellularly, neutrophils can release microbicidal and potentially toxic elements into the extracellular space through degranulation, which may exacerbate inflammatory tissue damage (7, 9).

Neutrophils also release lattices of decondensed chromatin decorated with antimicrobial granule proteins and degradative enzymes called neutrophil extracellular traps (NETs) into the extracellular space to trap and kill microbes (10). Neutrophils form NETs in response to many infectious and noninfectious stimuli (11, 12). However, excessive NET formation or decreased clearance of NETs from the inflammatory milieu promotes tissue damage in sepsis (13–15), arterial and venous thrombosis (16, 17), ischemia-reperfusion injury (18, 19), and may promote cancer growth and metastasis (20, 21). More recently, our group and others have reported pathogenic NET formation in patients with COVID-19 related immunothrombosis (22, 23) and experimental ischemic stroke (24).

Given the pathogenic potential of dysregulated NET formation, effective pharmacologic strategies targeting NET formation are needed (25), particularly in sepsis (26–28). We recently discovered a novel class of endogenous NET-inhibitory peptides generated by the placenta that circulate in the umbilical cord blood of fetuses and newborn babies and inhibit NET formation (12, 29). One such peptide is neonatal NET-Inhibitory Factor (nNIF). In our primary report, we observed that pretreatment with nNIF blocked NET formation and improved survival in mouse models of systemic inflammation and NET-mediated inflammatory tissue damage (12). However, such a pretreatment dosing regimen has limited clinical translatability as patients often present after sepsis has already begun. We, therefore, proposed to study NET-inhibition in a more clinically applicable model of experimental sepsis. Here we report that treatment with nNIF in a clinically relevant dosing regimen initiated 4 hours after cecal ligation and puncture (CLP), either as monotherapy or as an adjuvant to systemic antibiotic treatment, improves clinical illness severity and survival in the CLP model of experimental polymicrobial sepsis. Furthermore, increased survival following nNIF monotherapy compared to vehicle control resulted without changing the mouse peritoneal fluid and stool microbiomes as demonstrated by metagenomic analysis of peritoneal fluid and feces in this model of experimental sepsis.

## Materials and methods

### Animal studies

We purchased male outbred Swiss Webster mice, 8 weeks of life weighing 25-27 grams, from Charles River Laboratories. We housed our mice on a 12-hour light/12-hour dark cycle with a constant temperature in the University of Utah Center for Comparative Animal Studies in specific pathogen-free microisolator cages (5 mice/cage) with access to standard rodent food and water *ad libitum*. We weight-matched all mice at the initiation of experiments and randomized the mice to each treatment group. The researchers conducting the experiments were blinded to the experimental groups during testing including the clinical illness severity assessments. No inclusion or exclusion criteria were used in designing the experiments.

### Ethics statement

All murine experiments were approved by the University of Utah Institutional Animal Care and Use Committee (no. 21-09012) in the Center for Comparative Animal Studies at the University of Utah, which is approved by the American Association of Laboratory Animal Care.

### Reagents

We purchased lipopolysaccharide from *Salmonella enterica* serotype enteritidis (Sigma: L6011), poly-L-lysine (Sigma), SYTO Green (cell-permeable DNA stain; Molecular Probes), SYTOX Orange (cell-impermeable DNA stain; Molecular Probes), DNase 1 (Promega), micrococcal DNase (Worthington), isoflurane anesthesia from VETONE (FLURISO™), meropenem (Sargent^®^) from University of Utah Hospital, DMSO (Fisher Scientific: BP 231-1), and sterile isotonic saline (BD PosiFlush™;10 mL syringes).

### CLP model of sepsis and clinical illness severity assessment

Mice were anesthetized with 3% isoflurane in an induction chamber, and anesthesia was maintained by use of inhalation *via* nose cone. Sepsis was induced by cecal ligation and puncture (CLP) as previously described (30, 31). Vehicle (DMSO – 0.003%/5% Dextrose/H_2_0), nNIF (1 mg/kg in 0.003% DMSO/5% Dextrose/H_2_O) or nNIF-SCR (1 mg/kg in 0.003% DMSO/5% Dextrose/H_2_O), an inactive scrambled peptide control, was given intravenously (IV) in each of two total doses at 4 and 10 hours after CLP surgery. The animals received subcutaneous sterile isotonic saline (0.5 mL) for fluid resuscitation immediately after surgery. Sham-operated mice were subjected to identical procedures, except that ligation and puncture of the cecum was omitted. Survival of mice subjected to CLP or sham injury was determined daily for 6 days. Twenty-four and 48 hours after CLP, all animals were scored for clinical illness severity as previously described (32) by an examiner blinded to treatment arm. In this assessment, higher scores reflect increased illness severity with non-surviving mice receiving a score of 10. These mice were included in the statistical analysis. In additional experiments, mature Swiss-webster mice were treated with sham surgery or CLP procedure. At 4 hours after surgery, mice underwent blinded clinical illness severity assessment, measurement of body weight and temperature, peritoneal fluid and whole blood neutrophil counts, whole blood platelet counts, and plasma creatinine level determination.

### Experimental design and treatment

For our survival studies, animals were randomly assigned to one of five groups: (a) sham-operated group (Sham); (b) DMSO vehicle treatment group (CLP + DMSO (0.003%/5% Dextrose/H_2_O, IV); (c) nNIF treatment group (CLP + nNIF, 1 mg/kg, 0.003%/5% Dextrose/H_2_O, IV); (d) scrambled peptide control treatment group (CLP + nNIF-SCR, 1 mg/kg, 0.003%/5% Dextrose/H_2_O, IV); and (e) antibiotic treatment group (CLP + meropenem, 25 mg/kg, IP). Groups a-d received their respective treatment at 4 and 10 hours after CLP. Group e received two doses of meropenem at 4 and 10 hours after CLP and then daily injections for 6 days. In another set of survival studies, mice were treated with nNIF or nNIF-SCR (1 mg/kg, IV) 4 and 10 hours after CLP in combination therapy with a suboptimal dose of meropenem (8 mg/kg, IP) also given 4 and 10 hours after CLP and then once daily. Randomly selected animals were euthanized at 24 and 96 hours after CLP for sampling of blood and peritoneal fluid. Briefly, the blood of anesthetized mice was collected by cardiac puncture, following euthanasia. The peritoneal cavity was then opened and washed under aseptic conditions with 3 mL of cold PBS. Both aliquots of blood and peritoneal fluid were used for the following analyses: leukocyte cell count and differential, bacterial CFU studies, NET quantitation using MPO-DNA complex ELISA, live cell imaging, and multiplex cytokine profiling with specific ELISA confirmation.

### Live cell imaging of NETs

We evaluated mouse NET formation *via* live cell imaging with confocal microscopy as previously described (12). Sham mice and CLP animals from all experimental groups were euthanized by CO_2_ inhalation and the peritoneal fluid was collected. An aliquot of 100 μL of the peritoneal fluid was placed on glass coverslips coated with poly-L-lysine for 1 hour at 37°C in 5% CO_2_/95% air. NET formation was assessed using cell-permeable (SYTO Green, Molecular Probes) and cell-impermeable (SYTOX Orange, Molecular Probes) DNA fluorescent stains. Using a FV3000 1X81 confocal microscope and FluoView software (Olympus) with 20X and 60X objectives, we obtained z-series images over a range of 20 μm with a 1 μm step size for each field. We processed these images using FluoView (Olympus), Photoshop (Adobe), and ImageJ (NIH) software.

### MPO-DNA complex ELISA

An in-house ELISA was used to quantify MPO-DNA complexes in plasma and peritoneal fluid from sham and CLP mice 24 hours after the surgical procedure (24, 27). Briefly, after overnight coating with anti-MPO capture antibody in calcium carbonate coating buffer (2 µg/ml; 0400-0002, Bio-Rad) at 4°C, a 96-well plate was blocked with 2.5% bovine serum albumin in PBS for 2 hours at room temperature. The plate was subsequently washed, before incubating for 90 minutes at room temperature with 10% peritoneal fluid in blocking buffer. The plate was washed five times, and then incubated for 90 minutes at room temperature with anti-DNA detection antibody (1:20; Cell Death detection ELISA, 11544675001, Sigma). After five washes, the plate was developed with TMB substrate solution (Sigma).

### Cell counts from whole blood and peritoneal fluid

Twenty-four hours after CLP or sham surgery, total white blood cell and differential neutrophil counts were determined both in whole blood samples collected from orbital vein using heparinized capillaries tubes and peritoneal fluid by washing with 3 mL of PBS, respectively. Then, samples were incubated with CD45 APC-Cy7 (Biolegend), CD11b PE-Cy7 (EBioscience), Ly6G BV510 (Biolegend), and CD16/CD32 (Fc-block, EBioscience) for 30 minutes at 37^°^C. Thirty minutes later, cells were washed, fixed, and ran on a Beckman Coulter Cytoflex located in the University of Utah Flow Cytometry Core.

### Cytokine measurement and number of colony-forming units

Peritoneal fluid cytokines were analyzed using a mouse Cytokine/Chemokine Panel I Multiplex Array (Millipore Sigma catalog# HCYTMAG60-PMX41) according to manufacturer’s instructions on a Luminex 200 instrument. Bacterial counts in whole blood and peritoneal lavage were determined 24 hours after CLP. Briefly, peritoneal fluid and blood samples were collected, serially diluted, plated on LB-agar dishes, and incubated at 37°C for 24 hours. The number of bacterial colonies was counted and expressed as CFU/mL.

### 16S rRNA gene sequencing of peritoneal fluid and stool

Libraries were constructed for sequencing approximately 460 bp of the variable V3 and V4 regions of 16S rRNA using a two-step PCR process. Genomic DNA (5 to 20 ng) was PCR amplified for 14 cycles using the Quick-16S Primer Set V3-V4 (Zymo Research cat# D6405-2-400) with NEBNext Ultra II Q5 Master Mix (NEB cat# M0544). Dual unique indexing adapters (Illumina cat#20027213) were added to the amplicons during a second PCR reaction (6 cycles). 16S libraries were qualified on an Agilent Technologies 4150 TapeStation using a D1000 ScreenTape assay (cat# 5067-5582 and 5067-5583, respectively) and the molarity of adapter-modified molecules was defined by quantitative PCR using the Kapa Biosystems Kapa Library Quant Kit (cat#KK4824). Libraries were normalized and pooled (12 pM) and chemically denatured along with 15% molar representation of PhiX library in preparation for sequencing on an Illumina MiSeq instrument in paired-end 300 cycle mode.

Raw 16S sequences were read in and processed with QIIME2 v 2021.11 plugins (33). In brief, demultiplexed and quality-filtered sequences were first trimmed of primer and linker sequences with the Cutadapt plugin, then denoised to ASVs using DADA2 (34, 35). Taxonomies were then assigned to ASVs with the classify-sklearn method in the feature-classifier plugin, against the Greengenes 13_8 taxonomy. We then removed ASVs without at least a phylum level taxonomic assignment because we found that these sequences were mouse-derived and highly enriched in the peritoneal fluid samples. Diversity metrics, distances, and statistical significance of beta diversity groupings by permanova were further calculated within QIIME2 (36). QIIME2 artefacts were then read into R and genera tested for differential abundances using ANCOM-BC implemented in the microbiomeMarker package (37, 38). Scripts used for processing and analyses have been deposited along with processed QIIME2 artifact files at: https://github.com/wzacs1/Araujo_nNIF. Raw sequences have been deposited in the NCBI SRA under BioProject accession PRJNA884333.

### nNIF and scrambled peptide control synthesis

nNIF and its specific scrambled peptide control were synthesized by the DNA/Peptide Facility, a unit of the Health Sciences Center Cores at the University of Utah. The core facility also verified the sequence and purity of the provided peptides *via* mass spectroscopy.

### Statistics

We used GraphPad Prism software (version 9.4.1) for all statistical analyses. We used the Log-rank (Mantel-Cox) test for survival analysis with the Bonferroni correction for multiple comparisons. We used the two-tailed Student’s t-test for direct comparisons and the one-way ANOVA or Kruskal-Wallis statistical tools with appropriate *post hoc* testing for multiple comparisons depending on the normality of data distribution. For each experimental variable, we determined the mean ± SEM. All data used in each statistical test met the assumptions of the specific test. We considered P < 0.05 as statistically significant.

## Results

### CLP-treated mice demonstrate clinical illness and markers of early sepsis at 4 hours after surgery

**Figure 1:**
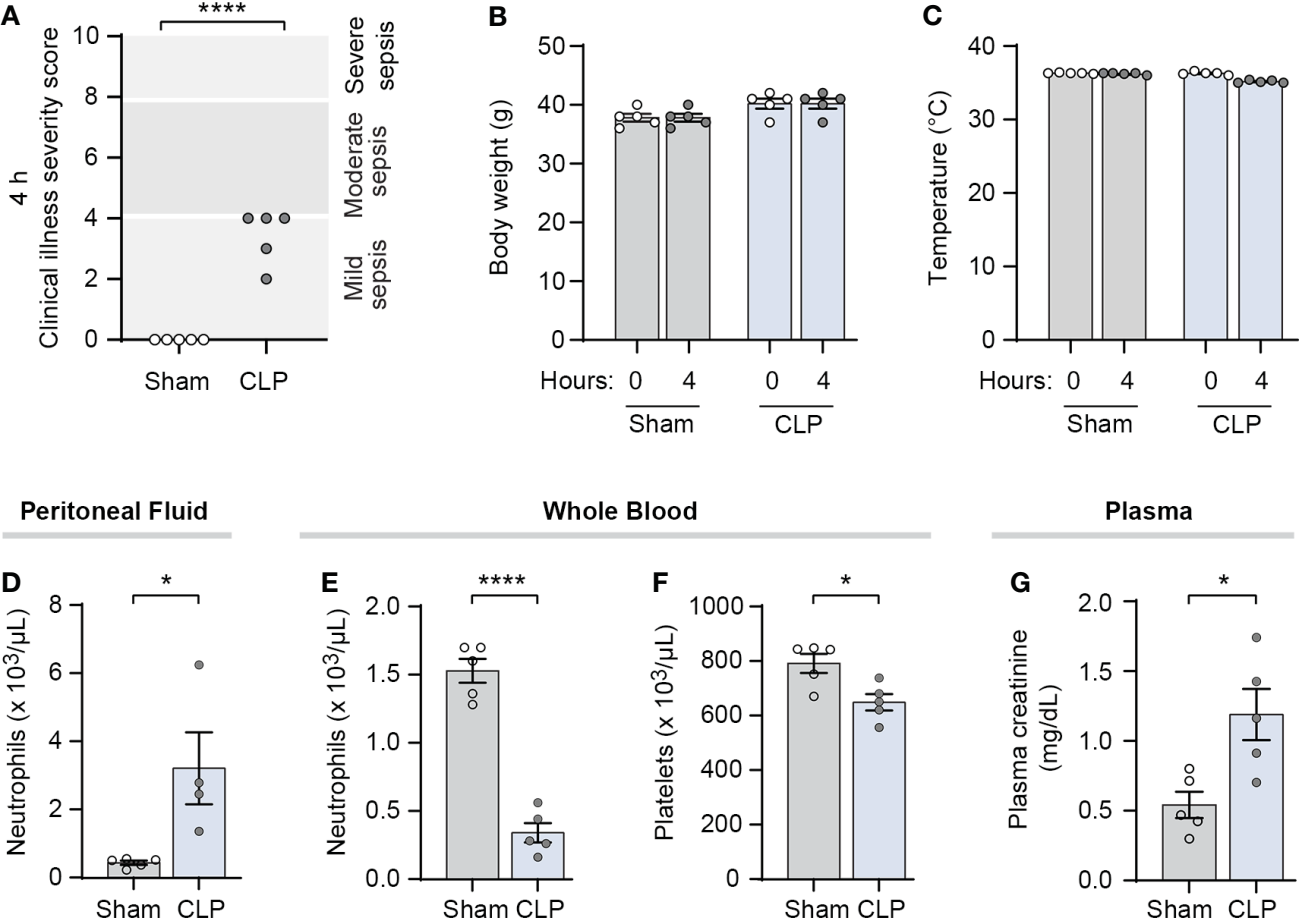
CLP-treated mice demonstrate clinical illness and markers of early sepsis at 4 hours after surgery. We performed CLP on mature outbred Swiss webster mice, with sham operated mice as controls (N=4-5 for both groups). At 4 hours after CLP, we assessed **(A)** clinical illness severity in a blinded fashion, **(B)** body weight before and after CLP or sham surgery, **(C)** temperature before and after CLP or sham surgery, **(D)** peritoneal fluid and **(E)** whole blood neutrophil counts, **(F)** whole blood platelet counts, and **(G)** plasma creatinine levels as markers of clinical illness. We used the student’s t-test statistical tool to compare sham and CLP treated mice in each assay. For this figure, * denotes P< 0.05 and **** denotes P<0.0001.

We characterized illness severity and markers for sepsis at the 4-hour timepoint in mature Swiss-webster mice following sham or CLP surgery. Markers of sepsis including cytokines and thrombocytopenia are known to be elevated in CLP-treated mice compared to sham surgery mice in multiple mouse strains including Swiss-webster mice (39–41). In our hands, we demonstrated increased illness severity scores at 4 hours in Swiss-webster mice treated with CLP compared to their sham-treated control mice (**Figure 1A**). We also found that while weight and temperature varied little during the first 4 hours after CLP (**Figures 1B, C**), peritoneal fluid neutrophil counts were significantly increased while peripheral blood neutrophil counts were significantly decreased (**Figures 1D, E**). In addition, CLP-treated mice had significantly lower platelet counts at 4 hours than did sham surgery controls (**Figure 1F**). Further, plasma creatinine levels were increased in CLP-treated mice compared to sham treated controls (**Figure 1G**). These results suggested the 4-hour timepoint in this CLP model as an appropriate time to initiate NET-inhibition with nNIF. Additional pharmacodynamic and pharmacokinetic studies performed in a different model, the LPS injection model of sterile inflammatory peritonitis, further suggested that nNIF’s NET-inhibitory effects would last through to a second dose given 6 hours after the first dose of nNIF in this model (**Supplemental Figures 1–3**).

### nNIF improves survival and illness severity in CLP-treated mice in a dose-dependent manner

**Figure 2:**
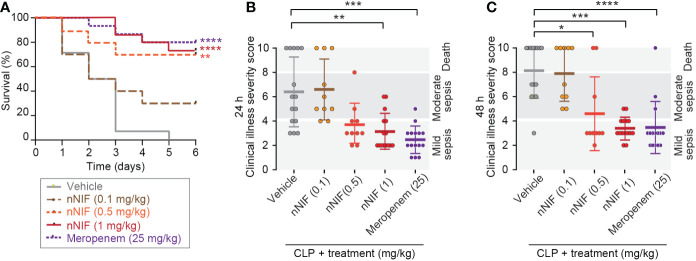
nNIF improves survival and illness severity in CLP-treated mice in a dose-dependent manner. We performed CLP on outbred Swiss webster mice ± nNIF in different doses (0.1-1 mg/kg, IV; N=10, 10, and 15 animals respectively in the nNIF groups), vehicle control (N=15), or meropenem (N=15) given at 4 and 10 hours after CLP. **(A)** Survival was assessed over 6 days. We used the Log-rank (Mantel-Cox) statistical tool to compare survival between all treatment groups. We also assessed sepsis illness severity 24 **(B)** and 48 hours **(C)** after CLP. We used the one-way ANOVA statistical tool with Newman-Keuls multiple comparison *post-hoc* testing to compare illness severity between all treatment groups. For this figure, * denotes P< 0.05, ** denotes P<0.01, *** denotes P<0.001, and **** denotes P<0.0001.

We previously reported that pre-treatment with nNIF or NIF-related peptides (NRPs) blocks NET formation, and consequently improves mortality in murine models of systemic inflammation (12). Here we sought to determine whether IV dosing of nNIF in a more clinically relevant regimen with two total doses of nNIF given at 4 and 10 hours after CLP surgery when signs of sepsis are evident (**Figure 1**) would provide a similar survival advantage. Using a dose-response curve of nNIF (0.1 – 1 mg/kg mouse body weight), we observed a significant improvement in survival in mice treated with nNIF at doses of 0.5 mg/kg (70%) and 1 mg/kg (73%) when compared to its vehicle (DMSO) control (0%) but minimal improvement in survival of mice who received the 0.1 mg/kg nNIF dose (30%) (**Figure 2A**). Given these results, we chose the nNIF dose of 1 mg/kg for subsequent experiments. As expected, mice treated with the antibiotic meropenem (25 mg/kg, IP) also showed an improvement in survival (80%). No mortality was observed in sham-operated animals (data not shown). In addition, the protective effect of nNIF on survival also correlated with improvement in the illness severity scores at 24 hours (**Figure 2B**) for the 1 mg/kg dose and at 48 hours (**Figure 2C**) for the 0.5 mg/kg and 1 mg/kg doses tested as compared to the vehicle control.

### nNIF, but not meropenem, decreases peritoneal NET formation and neutrophil CD11b expression without altering peritoneal WBC and neutrophil counts in CLP mice

**Figure 3:**
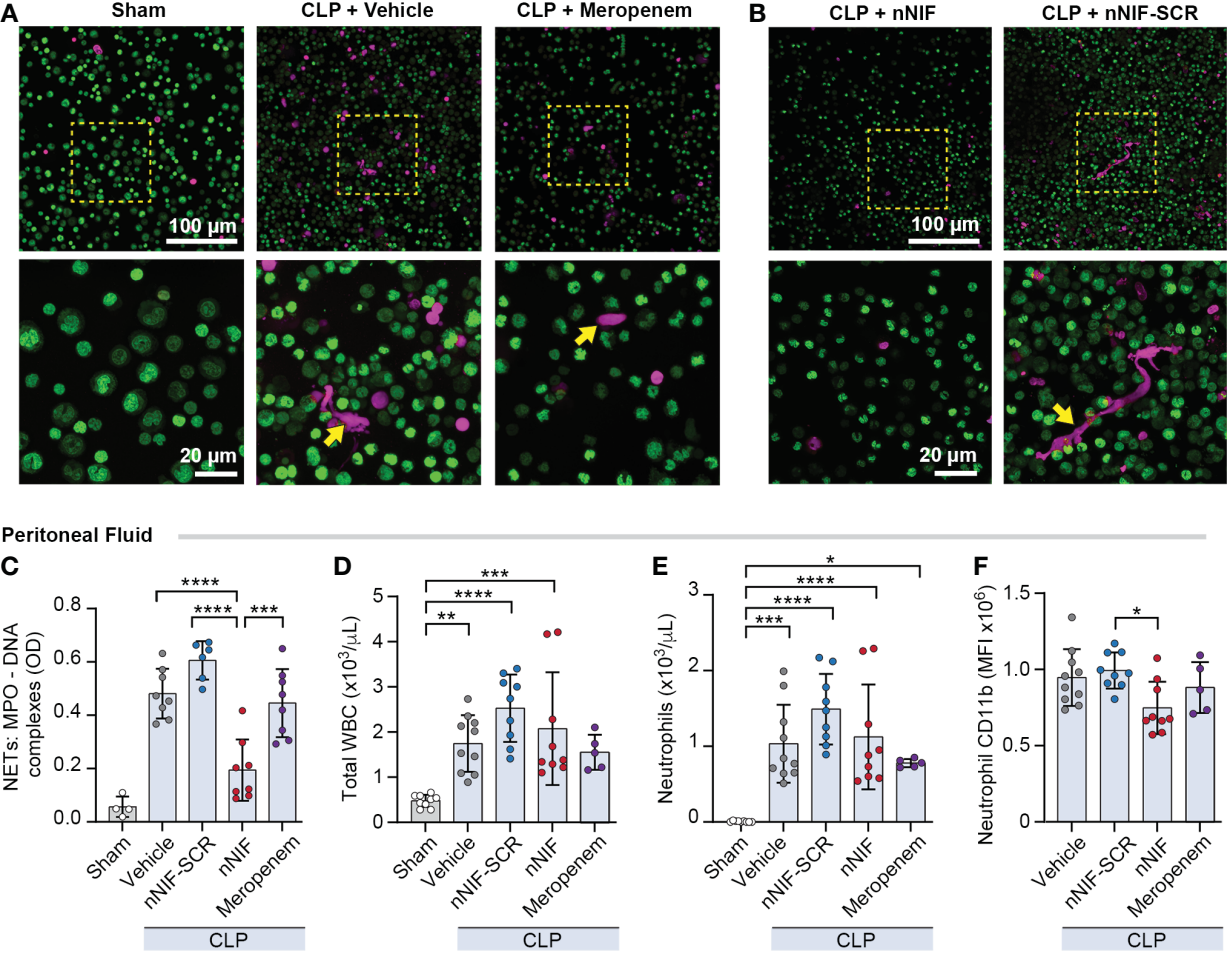
nNIF, but not meropenem, decreases peritoneal NET formation and neutrophil CD11b expression without altering peritoneal WBC and neutrophil counts in CLP mice. We assessed peritoneal NET formation in CLP treated mice ± nNIF (1 mg/kg), nNIF-SCR (1 mg/kg), or meropenem (25 mg/kg) given 4 and 10 hours after CLP. We detected NET formation qualitatively **(A, B)** via confocal microscopy using a cell-impermeable DNA dye (red). Nuclear DNA was detected using a cell permeable DNA dye (green). 20x and 60x magnification images are shown. Representative images of 4 different mice in each of 2 different experiments. **(C)** We quantified peritoneal fluid NET formation 24 hours after CLP in all treatment groups using the MPO-DNA ELISA to determine levels of MPO-DNA complexes as a surrogate for NET formation. **(D-F)** We performed flow cytometry on peritoneal fluid samples 24 hours after CLP to determine **(D)** total white blood cell counts (WBC), **(E)** neutrophil counts, and **(F)** neutrophil CD11b expression in all treatment groups. N= 5-9 mice per group. We used the One-way ANOVA statistical tool with Newman-Keuls multiple comparison post hoc testing. * denotes P< 0.05, ** denotes P<0.01, *** denotes P<0.001, and **** denotes P<0.0001.

We next determined whether nNIF inhibits CLP-induced peritoneal NET formation. We collected peritoneal fluid by lavage at 24 hours after CLP. Using confocal microscopy following CLP, we identified robust cell free DNA release from peritoneal leukocytes and confirmed that there was increased NET release using the MPO-DNA ELISA. Treatment with nNIF following CLP reduced NET formation qualitatively and quantitatively compared to vehicle control, scrambled peptide control, and meropenem treated mice. Sham operated mice did not have peritoneal NET formation (**Figures 3A–C**). We next examined the effect of nNIF treatment on leukocyte infiltration in the peritoneal cavity after CLP. At 24 hours following CLP, and in all groups except meropenem, we observed a significant increase in total white blood cell numbers in the peritoneal cavity compared to sham mice (**Figure 3D**). With no significant differences noted between nNIF and the scrambled peptide control, this effect was rightly attributed to an effect of CLP alone. We also observed elevated peritoneal fluid neutrophil counts in all groups when compared to the sham surgery group (**Figure 3E**). Finally, as a marker for bacterial infection, we analyzed neutrophil CD11b integrin expression 24 hours after CLP (42). We observed significantly decreased neutrophil CD11b expression in the peritoneal fluid of nNIF-treated CLP mice compared to the scrambled peptide control-treated CLP mice but no decrease in meropenem treated mice. Additionally, neutrophil CD11b expression was detected in sham-treated mice; however, in comparison to neutrophil CD11b expression in vehicle-treated CLP mice, the levels were significantly lower (**Figure 3F**; data not shown).

### nNIF, but not meropenem, decreases systemic NET formation without affecting white blood cell or neutrophil counts in mice following CLP

**Figure 4:**
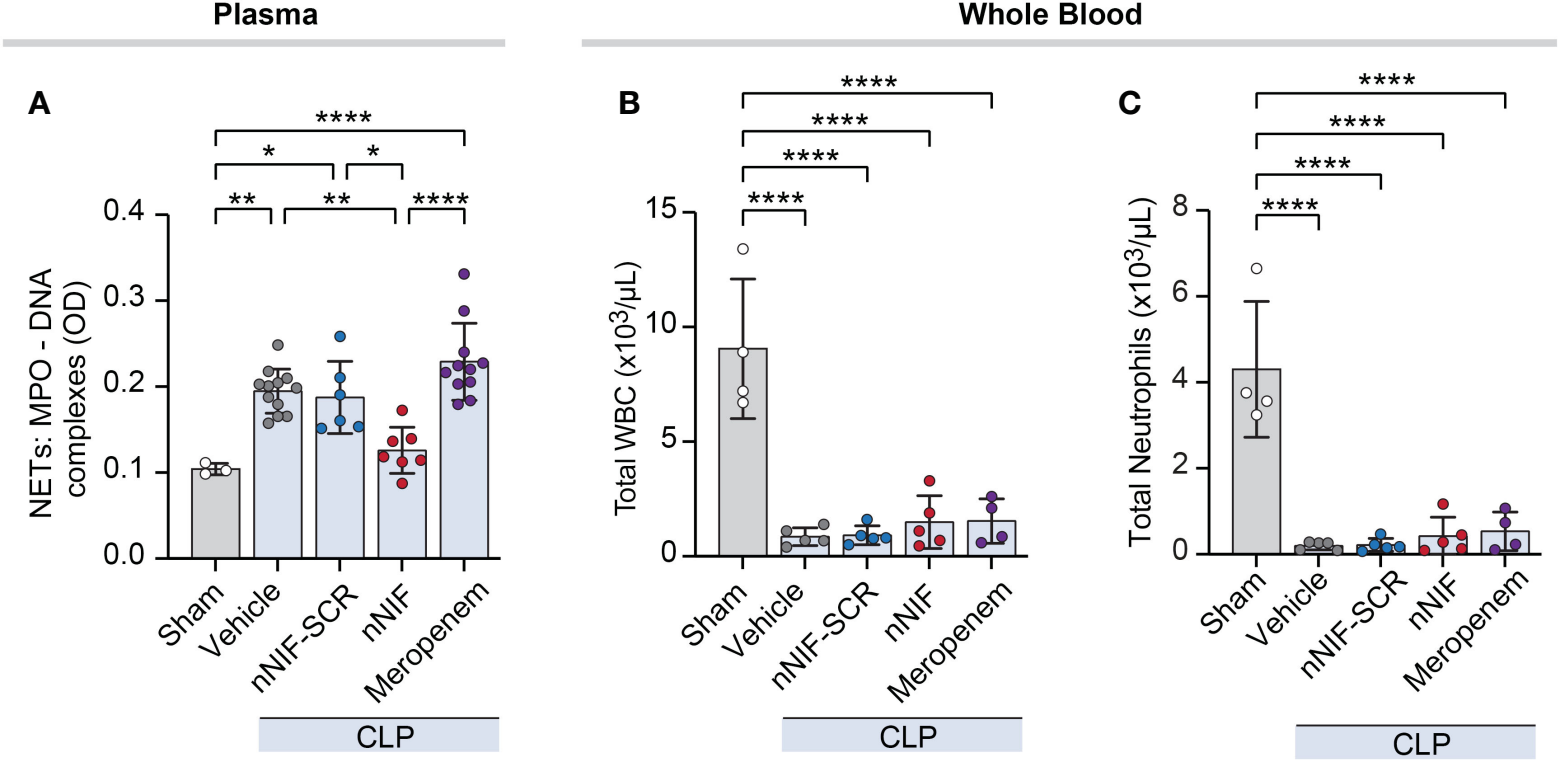
nNIF, but not meropenem, decreases systemic NET formation without affecting white blood cell or neutrophil counts in mice following CLP. We assessed plasma NET levels and leukocyte counts in mature mice ± nNIF (1 mg/kg), nNIF-SCR (1 mg/kg), meropenem (25 mg/kg), or vehicle control given 4 hours and 10 hours after CLP. **(A)** We measured plasma MPO-DNA complex levels as a surrogate for NET formation 24 hours after CLP using the MPO-DNA complex ELISA. **(B, C)** We analyzed leukocyte and neutrophil cell counts in whole blood using flow cytometry 24 hours after CLP. **(B)** Total white blood cells (WBC), **(C)** total neutrophils. N = 3-5 different mice per group in two separate experiments. We employed the One-way ANOVA statistical tool with Newman-Keuls multiple comparisons post hoc testing. * denotes P< 0.05, ** denotes P<0.01, and **** denotes P<0.0001.

We next quantified MPO-DNA complexes in mouse plasma at 24 hours after surgery in CLP-treated mice ± nNIF treatment. We observed significantly increased MPO-DNA complexes in peripheral blood of CLP mice treated with vehicle, scrambled peptide control, or meropenem, but not nNIF compared to the sham surgery group (**Figure 4A**). Furthermore, nNIF-treated animals showed a significant reduction in MPO-DNA complexes compared to vehicle-treated, scrambled peptide control-treated, and meropenem-treated CLP mice at 24 hours (**Figure 4A**). We also observed significantly decreased numbers of both total white blood cells and neutrophils in the peripheral blood of vehicle, scrambled peptide control, nNIF, and meropenem treated mice when compared to sham surgery mice (**Figures 4B, C**).

### NET inhibition with nNIF increases peritoneal fluid and blood aerobic bacterial colony forming units and alters peritoneal fluid and stool microbiomes compared to the vehicle control group

**Figure 5:**
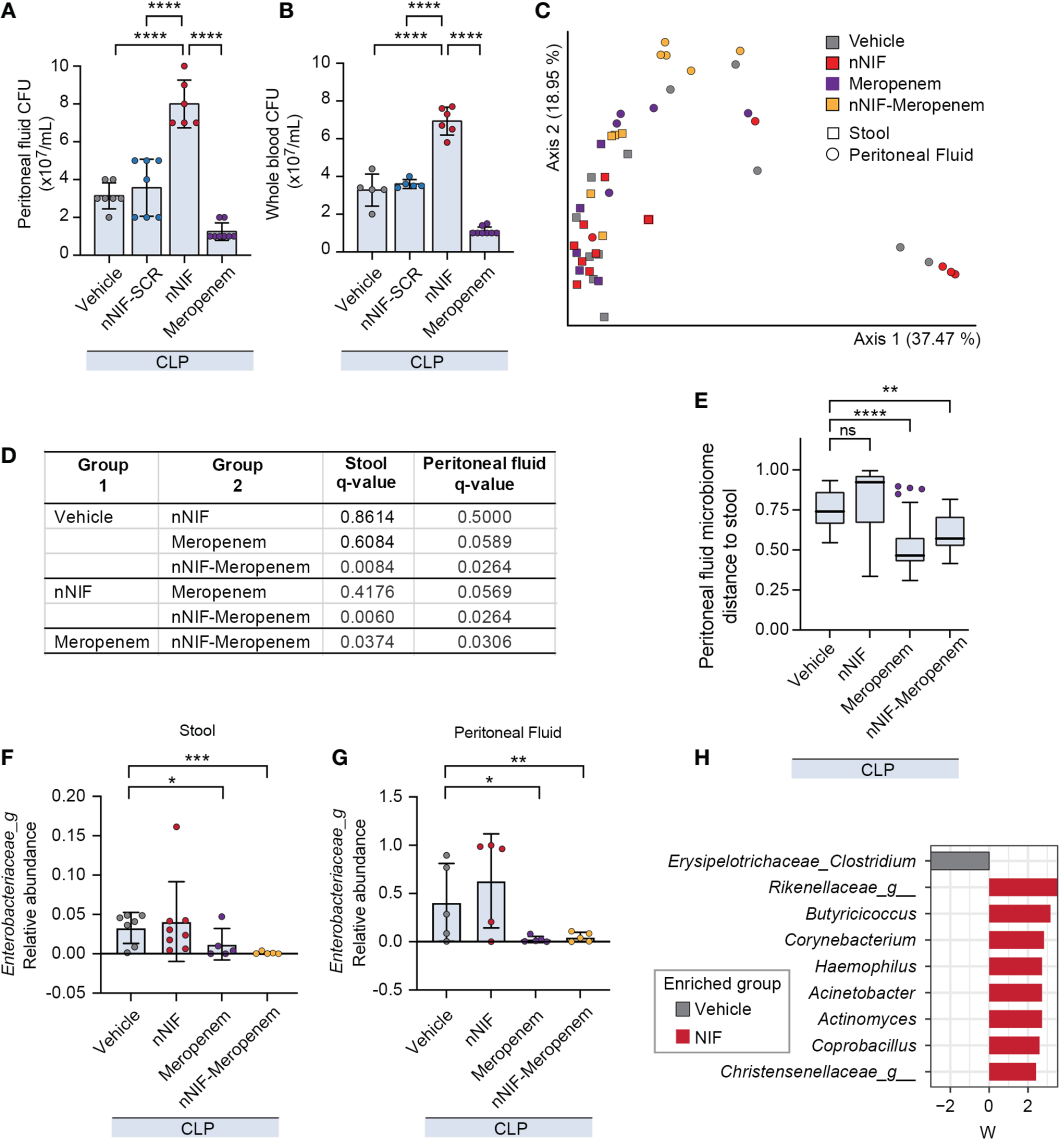
NET inhibition with nNIF increases peritoneal fluid and blood aerobic bacterial colony forming units (CFUs) and alters peritoneal fluid and stool microbiomes compared to the vehicle control group. We assessed bacterial colony forming units via serial dilution in peritoneal fluid and whole blood as well as beta diversity of the stool and peritoneal fluid microbiomes of CLP-treated mice in the following treatment groups: sham, vehicle control, meropenem alone, nNIF alone, and nNIF-meropenem combined. Metagenomic data was generated using 16S rRNA gene sequencing of the V3/V4 hypervariable region. **(A, B)** We determined bacterial colony forming units in **(A)** peritoneal fluid and **(B)** whole blood via serial dilution and culture. N=5-8 samples per group. We employed the one-way ANOVA statistical tool with Tukey’s multiple comparisons post hoc testing. **** denotes P<0.0001. **(C)** Principal coordinates plot and **(D)** PERMANOVA results of stool and peritoneal fluid microbiomes based on Bray-Curtis Distance between pairs. N=5-7 in each group. **(E)** Bray-Curtis distance from stool for peritoneal fluid samples from each group (y-axis). Significance was determined by Kruskal-Wallis with Dunn’s multiple comparison test comparing each group to vehicle. **(F, G)** Relative abundances of Enterobacteriaceae in stool **(F)** and peritoneal fluid **(G)** microbiomes. Significant differences relative to vehicle treatment only are shown and reflect adjusted p values determined by ANCOM-BC. **(H)** Differentially abundant bacterial genera in peritoneal fluid due to nNIF treatment compared to vehicle as determined with ANCOM-BC. Bacterial genera differentially abundant are shown on the y-axis with ANCOM-BC statistic on the x-axis. * Denotes P< 0.05, ** denotes P<0.01, *** denotes P<0.001. ns denotes a difference not statistically significant.

We next evaluated the bacterial load in the peritoneal cavity and peripheral blood of CLP mice treated with nNIF, nNIF-SCR, or meropenem. As expected, the number of aerobic bacterial CFUs was higher both in the peritoneal fluid and blood in vehicle-treated CLP mice compared to the sham surgery mice at 24 hours after CLP (data not shown). Likewise, mice treated with nNIF exhibited higher CFUs in peritoneal fluid and peripheral blood compared to the vehicle-treated and scrambled peptide control treated mice (**Figures 5A, B**), further supporting nNIF as a potent inhibitor of NET-mediated extracellular microbial killing. As expected, the mice treated with meropenem showed a statistically significant decrease in both peritoneal fluid and peripheral blood bacterial CFUs as compared to all other experimental groups (**Figures 5A, B**). These results suggested potential alterations in the peritoneal fluid and stool microbiomes following NET inhibition. Using sequencing of the V3/V4 regions of the bacterial 16S rRNA gene we determined peritoneal fluid and stool microbiomes in CLP-treated mice ± nNIF, meropenem, or combination therapy of the two. We demonstrated significantly different peritoneal fluid and stool microbiomes in nNIF-meropenem treated CLP mice as compared to all other experimental groups (**Figures 5C, D**). We also demonstrated that peritoneal fluid microbiomes with nNIF treatment alone were as dissimilar from their stool counterparts as were vehicle controls, while meropenem treatments yielded more similar stool and peritoneal microbiomes (**Figure 5E**). Further, we showed that, as expected, the Enterobacteriaceae were at higher relative abundance in peritoneal fluid and were decreased in stool and peritoneal fluid only with meropenem containing treatments, while nNIF alone did not affect their abundances (**Figures 5F, G**). nNIF treatment alone resulted in an increase in peritoneal fluid CFUs of common mammalian gut commensal bacteria genera and a relative decrease in one genus-unclassified family of commensal Clostridium, the Erysipelotrichaceae (**Figure 5H**).

### NET inhibition with nNIF decreases TNF-α levels in peritoneal fluid in CLP-treated mice

**Figure 6:**
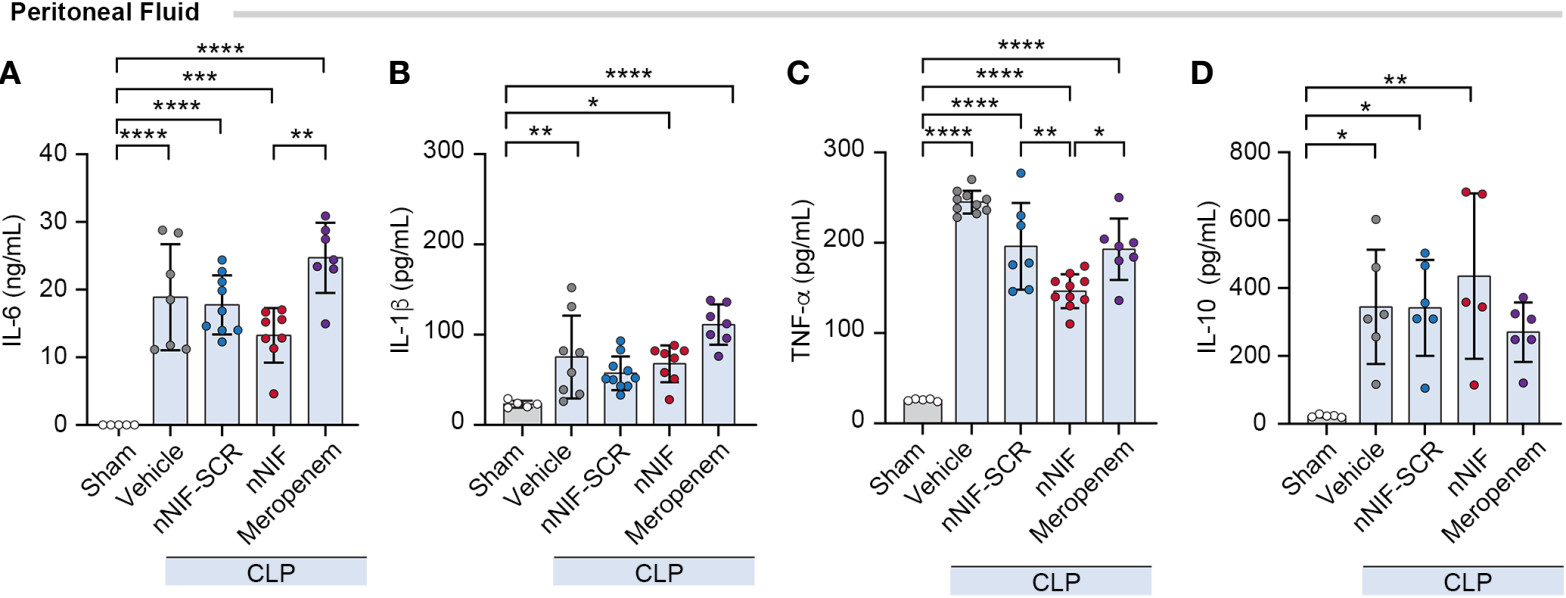
NET inhibition with nNIF decreases TNF-α levels in peritoneal fluid in CLP-treated mice. Using a Luminex multiplex ELISA, we determined **(A-D)** peritoneal fluid cytokine levels – IL-6, IL-1β, TNFα, and IL-10. Mean ± SEM shown for N = 5-9 separate mice per group in 2 separate experiments. We employed the One-way ANOVA statistical tool with Tukey’s multiple comparisons *post hoc* testing. * Denotes P<0.05, ** denotes P<0.01, *** denotes P<0.001, **** denotes P<0.0001.

We next investigated the potential anti-inflammatory effect of nNIF treatment in CLP mice by analyzing the levels of cytokines expressed in the peritoneal fluid at 24 h after CLP. We observed an increase in both pro-inflammatory (IL-6, IL-1β, TNF-α) and anti-inflammatory (IL-10) cytokine levels after CLP surgery when compared to the sham group (**Figures 6A–D**). However, when the mice were treated with nNIF, we observed decreased TNF-α levels in peritoneal fluid compared to all other treatment groups, while IL-6 levels were decreased compared to meropenem treated mice but not compared to the nNIF-SCR treatment group. Other cytokine level comparisons showed significant increases compared to sham treated mice, but comparisons between treatment groups showed no other differences (**Figures 6A–D**).

### Adjunctive nNIF treatment with sub-optimally dosed meropenem improves survival and illness severity in CLP-treated mice

**Figure 7:**
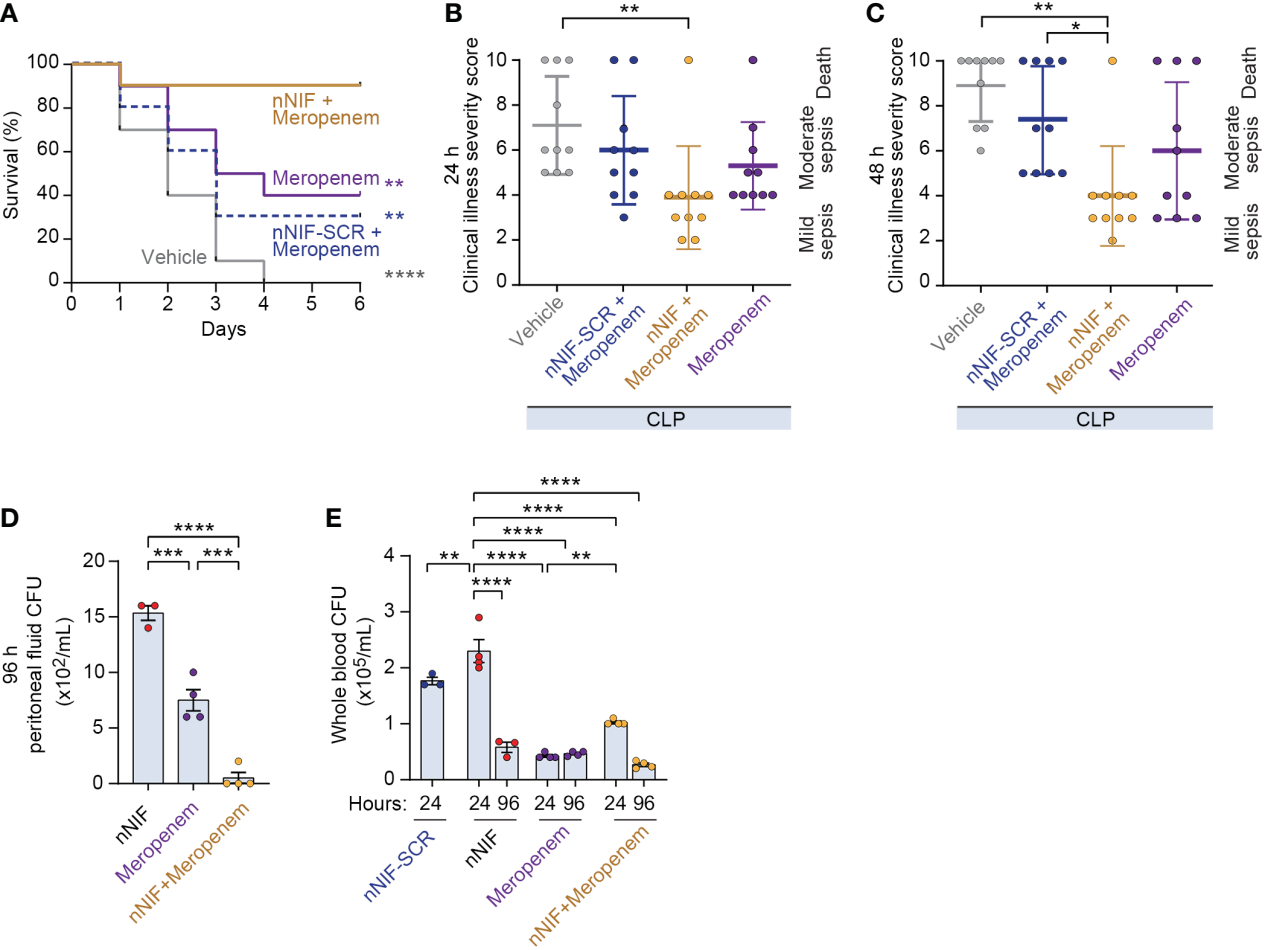
Adjunctive nNIF treatment with sub-optimally dosed meropenem improves survival and illness severity in CLP-treated mice. We assessed survival and clinical illness severity in CLP-treated mice receiving meropenem (8 mg/kg/dose) 4 and 10 hours after CLP ± nNIF (1 mg/kg), nNIF-SCR (1 mg/kg), or vehicle control given 4 and 10 hours after CLP. **(A)** Percent survival is shown on the y-axis. Survival curves for vehicle control (solid gray), meropenem alone (solid purple), meropenem-nNIF-SCR (dashed blue), and meropenem-nNIF (solid gold) are shown. N = 10 separate mice per group. We used the Log-rank (Mantel-Cox) statistical tool to compare survival between groups. **(B, C)** We tracked illness severity scores at 24 and 48 hours after CLP. Illness severity is shown on the y-axis at **(B)** 24 and **(C)** 48 hours after CLP. **(D, E)** We determined bacterial colony forming units in **(D)** peritoneal fluid and **(E)** whole blood *via* serial dilution and culture. N=3-4 samples per group. We employed the one-way ANOVA statistical tool with Tukeys or Newman-Kuels multiple comparisons *post hoc* testing. For this figure, * denotes P< 0.05, ** denotes P<0.01, *** denotes P<0.001, **** denotes P<0.0001.

To determine whether nNIF might be useful as an adjuvant therapy to antibiotic treatment in the setting of sepsis, we performed a meropenem dose response experiment and determined that an intraperitoneal meropenem dose of 8 mg/kg conferred a 40% survival rate (**Supplemental Figure 4**). We next used the 8 mg/kg meropenem dose to evaluate survival in CLP-treated mice ± nNIF, scrambled peptide control, or vehicle control. All vehicle control CLP-treated mice died by 4 days. Only 30% of the mice treated with the scrambled peptide control and the sub-optimal dose of meropenem survived over 6 days (**Figure 7A**). However, treatment with nNIF in combination with the 8 mg/kg dose of meropenem improved survival to 90% (**Figure 7A**). In these experiments, adjuvant nNIF with 8 mg/kg meropenem treatment also improved clinical illness severity at 24 and 48 hours after CLP compared to vehicle controls (**Figures 7B, C**). Finally, we assessed peritoneal and whole blood CFU levels at 24 and 96 hours after CLP in mice ± nNIF, scrambled peptide control, meropenem (8 mg/kg), or meropenem (8 mg/kg) + nNIF treatments. We found that in the peritoneal fluid at 96 hours, the nNIF-meropenem group demonstrated decreased bacterial CFU counts compared to the nNIF and meropenem treatment groups (**Figure 7D**). However, whole blood bacterial CFUs in nNIF treated mice, while elevated compared to all treatment groups at 24 hours, returned to meropenem alone treatment levels by 96 hours after CLP (**Figure 7E**). As expected, neither mortality nor clinical signs of sepsis were observed in sham surgery group at 24, 48, or 96 hours after CLP surgery (data not shown).

## Discussion

NETs serve as an innate defense mechanism responsible for pathogen clearance, but NETs may also induce collateral damage to host tissues. Thus, the regulation of NET formation with regard to location, timing, and magnitude is critical to efficient microbial containment while limiting host damage (43). One of the most severe examples of NET injury is immunothrombosis leading to multiorgan failure and death in sepsis (44, 45). In fact, patients with sepsis often face high mortality once the inflammatory process has begun with limited treatment options to change outcomes. Further insight into the pathogenicity of sepsis and new targets could offer novel therapeutic options for clinicians treating this often-deadly condition.

The current investigation shows that treatment with nNIF after the onset of sepsis leads to a sustained reduction of NETs with improved clinical outcome in a murine model of polymicrobial sepsis. We demonstrated that nNIF administration was highly effective in improving both survival and the illness severity score of mice when given after early sepsis had already begun (39–41). Qualitive measurement of increased DNA fluorescence with confocal microscopy combined with quantitative measurement of NET formation with MPO-DNA ELISA revealed a significant reduction of NETs in both the plasma and peritoneal lavage fluid of septic mice. Importantly, our data highlight the potential role of nNIF and NET inhibition as a new treatment approach in the early phase of infection in polymicrobial sepsis *in vivo.* This study corroborates findings from our recent study in which nNIF treatment before or after initiation of peritonitis inhibited NET formation in a murine model of neonatal polymicrobial peritonitis and endotoxemia resulting from intraperitoneal LPS injection (27). In the current study, we go a step further and investigate nNIF as an adjuvant therapy in combination with a broad-spectrum antibiotic for the treatment of septic mice. We included an antibiotic in this study to reproduce the common treatment paradigm used in septic patients and to determine if combined therapy could improve outcomes.

We found that nNIF improves survival in a dose-dependent manner in mice with CLP. Furthermore, we showed that the clinical illness severity score for the mice treated only with nNIF at both 24 and 48 hours were reduced to levels approaching CLP mice treated with meropenem, a broad-spectrum antibiotic. Our results demonstrated, as predicted, that nNIF (1 mg/kg) given at 4 and 10 hours after CLP surgery reduced the formation of NETs after sepsis onset. We measured NET levels by both MPO-DNA ELISA and confocal imaging, increasing the confidence of our findings of decreased NETs following nNIF delivery. The dose-dependent decrease in survival that we observed with decreasing doses of nNIF offers further evidence that NETs play an important role in the poor outcome in sepsis. Thus, our mouse model data suggest that a NET inhibitor, such as nNIF, delivered to a patient after the onset of sepsis has the potential to decrease NET levels and lead to reduced clinical illness severity and possibly even improved survival.

Twenty-four hours after nNIF administration, we measured neutrophil CD11b expression in peritoneal fluid of mice from each treatment group. Increased neutrophil CD11b expression levels are proposed as a potential early warning marker to detect neonatal bacterial infection and to predict death, although the literature is mixed (42, 46). This neutrophil cell surface antigen increases substantially within a few minutes after the cell encounters bacteria or endotoxins (47). As expected, the neutrophil surface expression of CD11b in the peritoneal fluid was increased in our CLP model and, surprisingly, the nNIF treated group had significantly decreased neutrophil CD11b expression compared to the scrambled peptide control but not compared to the meropenem treated animals. We postulate that neutrophil CD11b kinetics may be altered by nNIF acting as an anti-inflammatory agent.

We also demonstrated decreased total WBC and neutrophil counts along with decreased NETs in the peritoneal fluid of nNIF-treated mice at 24 hours after CLP. When considering the low DNA staining on confocal imaging of peritoneal leukocytes, it may be that the NET inhibition contributes to decreased endothelial dysfunction (48) and prevents immunothrombosis (22) which then leads to decreased inflammation. Alternatively, or in combination, the lack of NETs may lead to decreased opportunities to capture bacteria and thereby limit the chance that circulating PMNs will recognize and become activated by bacterial pathogens. This is supported by the fact that nNIF-treated mice displayed a rapid and considerable increase of CFU counts at the site of infection in the peritoneal fluid as well as in the plasma as opposed to the bacteria being removed from circulation by NETs. Of note, we previously demonstrated that neutrophils exposed to nNIF still maintain their ability to generate reactive-oxygen species and phagocytize *E. coli* bioparticles (12, 27). Considering all the data together, we propose that NET inhibition with nNIF effectively blocks NET formation leading to increased bacterial CFUs due to lack of NET-mediated extracellular microbial killing and decreased neutrophil activation. However, the ability of nNIF to leave neutrophil function intact may explain why nNIF-treated mice demonstrated decreased whole blood bacterial CFU levels at 96 hours compared to CFU levels at 24 hours after CLP, with 96-hour levels comparable to meropenem treated mice assessed in parallel. This may also partially explain the remarkable survival of nNIF treated mice even with decreased extracellular NET-mediated microbial killing. The finding of greater than 70% survival in CLP mice even with increased CFU counts demonstrates the potential therapeutic importance of blocking NET formation in sepsis.

An important part of our study was to test whether NET inhibition combined with broad spectrum antibiotic use would impact both bacterial microbiome and mouse survival. Meropenem is clinically used for intraperitoneal infections unresponsive to initial empiric therapy and was selected for these experiments to replicate a clinical treatment strategy. In our CLP models, we found that high dose of meropenem (25 mg/kg, IP) as monotherapy achieved over 80% survival in septic mice. However, meropenem did not reduce NETs and the MPO-DNA levels in the peritoneal fluid and plasma were equivalent between the vehicle and meropenem arms. Subtherapeutic doses of meropenem (8 mg/kg, IP) led to a decrease in survival to 40% with increased clinical illness severity scores in the CLP mice. However, when we combined the subtherapeutic meropenem dose with nNIF (1 mg/kg, IP), we achieved 90% survival with decreased clinical illness severity scores for both 24 and 48 hours. The ability of nNIF to almost double survival in suboptimal antibiotic dosing further supports the importance of NET inhibition to optimize survival in the setting of sepsis. Reassuringly, nNIF treatment alone did not change the dissimilarity of peritoneal fluid microbiome compared to the stool microbiome, while meropenem treatment did, and had only minor effects on the proportions of a few gut commensals genera found in peritoneal fluid during the CLP model. This is encouraging as empiric antibiotic treatment can lead to pathogenic bacterial overgrowth such as *C. difficile* that increase morbidity for patients. If lower doses of broad-spectrum antibiotics can be administered to patients in combination with nNIF, then it may be possible to avoid some of these iatrogenic issues that exacerbate morbidity in patients with sepsis.

One of the intriguing findings from our study is the decrease in peritoneal fluid TNF-α levels compared to nNIF-SCR, meropenem, and vehicle treated groups. Low sample size may have led to our inability to demonstrate significant decreases in IL-6 peritoneal fluid levels in this study. However, these data fit with the sepsis model of NETosis leading to immunothrombosis with subsequent systemic inflammation and cytokine storm. By reducing NET formation, nNIF may break this cycle of inflammation by avoiding immunothrombosis with accompanying multiorgan failure. IL-6 and TNF-α represent potent proinflammatory cytokines which correlate with increased mortality and morbidity in recent studies of pediatric and adult sepsis (49–52). Whether the cytokine-specific effects of nNIF coupled with direct TNF-α or IL-6 inhibitors would yield clinical benefits remains unknown. Of particular interest is the lack of an nNIF-associated alteration in IL-10 levels in this experimental model; IL-10 plays a prominent role in suppressing pro-inflammatory cytokine elaboration and represents a potent anti-inflammatory cytokine response to inflammation (53). Thus, nNIF may decrease the NET-associated cytokine storm in sepsis while not hindering or potentiating the immune suppressive components of the immune response to infection.

Additionally, we report for the first-time significant aspects of nNIF’s pharmacodynamic (PD) and pharmacokinetic (PK) properties (**Supplemental Figures 1–3**). nNIF provides peritoneal NET-inhibitory effects when given up to 8 hours intravenously before an inflammatory challenge and in our model that correlated with a time-dependent decrease in survival in the interval between 6 and 12 hours for nNIF dosing prior to injection of LPS. Pharmacokinetic data show an initial rapid decrease in nNIF plasma concentrations followed by a slower rate of decay (**Supplemental Figure 3**). While the pharmacokinetic data presented were generated using a higher nNIF dose (10 mg/kg) than employed in these experiments and in a different model of peritonitis, these data did inform our nNIF treatment regimen for these studies, and we hypothesize either that nNIF is effective at a lower concentration than our PK assay could detect (< 3 nM), which would be consistent with our previous report (12), or that the peptide is rapidly taken up by neutrophils and perhaps other extracellular trap forming myeloid leukocytes where it exerts its NET inhibitory effects over time or potentially for the life to the circulating leukocyte. Additional PD and PK data are now being generated as part of nNIF’s drug development for the clinic (Peel Therapeutics, SLC, Utah).

In conclusion, we demonstrate for the first time that nNIF given in combination with subtherapeutic doses of meropenem increases survival to 90% in mice with CLP-induced sepsis. This increase in survival occurs despite an increase in bacterial CFUs in the peritoneal fluid and blood of nNIF treated mice. nNIF offers the therapeutic advantage of maintaining WBC counts and neutrophil function while blocking NET release and subsequent cytokine activation. We hypothesize that the decreased TNF-α levels we observed in nNIF treated mice reflect decreased endothelial damage and lack of immunothrombosis due in part to NET inhibition. This lowered inflammatory state may attenuate the multiorgan failure often seen in sepsis and explain, in part, the decreased clinical illness severity scores in nNIF treated mice. When given after the onset of illness, nNIF may offer a new therapeutic modality to reduce morbidity and improve survival in sepsis and other NET-related inflammatory diseases.

## Data availability statement

The raw data presented in the study are deposited in the NCBI repository, accession number PRJNA884333. Scripts used for processing and analyses have been deposited along with processed QIIME2 artifact files at: https://github.com/wzacs1/Araujo_nNIF.

## Ethics statement

The animal study was reviewed and approved by University of Utah Institutional Animal Care and Use Committee (no. 21-09012).

## Author contributions

CY and CA conceived the project and designed the experiments. CA, FD, MC, JC, AP, KQ, JR, and JE performed experiments and analyzed data. JF, JE, and MK developed and performed the NET-Inhibitory Peptide quantitative assay. WS and QL performed the metagenomic sequencing, analysis, and dataset sharing. RC, JS, and CY wrote and edited the manuscript for important intellectual content. All authors contributed to the article and approved the submitted version.
