# Corrigendum: Neonatal NET-Inhibitory Factor improves survival in the cecal ligation and puncture model of polymicrobial sepsis by inhibiting neutrophil extracellular traps

**DOI:** 10.3389/fimmu.2023.1171222

**Published:** 2023-03-29

**Authors:** Claudia V. de Araujo, Frederik Denorme, W. Zac Stephens, Qing Li, Mark J. Cody, Jacob L. Crandell, Aaron C. Petrey, Kimberly A. Queisser, John L. Rustad, James M. Fulcher, Judah L. Evangelista, Michael S. Kay, Joshua D. Schiffman, Robert A. Campbell, Christian C. Yost

**Affiliations:** ^1^ Department of Pediatrics/Neonatology, University of Utah, Salt Lake City, UT, United States; ^2^ Molecular Medicine Program, University of Utah, Salt Lake City, UT, United States; ^3^ Department of Pathology, University of Utah, Salt Lake City, UT, United States; ^4^ High Throughput Genomics and Bioinformatic Analysis Shared Resource, Huntsman Cancer Institute, University of Utah, Salt Lake City, UT, United States; ^5^ Department of Biochemistry, University of Utah, Salt Lake City, UT, United States; ^6^ Department of Pediatrics/Hematology-Oncology, University of Utah, Salt Lake City, UT, United States; ^7^ Peel Therapeutics, Inc., Salt Lake City, UT, United States; ^8^ Department of Internal Medicine, University of Utah, Salt Lake City, UT, United States

**Keywords:** sepsis, neutrophil, neutrophil extracellular trap, neonatal NET-Inhibitory Factor, cecal ligation and puncture, microbiome, antibiotic resistance, innate immunity

In the published article, there was an error in the article title. Instead of “Neonatal NET-Inhibitory Factor improves survival in the cecal ligation and puncture model of polymicrobial by inhibiting neutrophil extracellular traps”, it should be “Neonatal NET-Inhibitory Factor improves survival in the cecal ligation and puncture model of polymicrobial sepsis by inhibiting neutrophil extracellular traps”.

In the published article, there was an error in the author list, and author James M. Fulcher was erroneously excluded. The corrected author list appears below:

Claudia V. de Araujo^1,2^, Frederik Denorme^2^, W. Zac Stephens^3^, Qing Li^4^, Mark J. Cody^1,2^, Jacob L. Crandell^2^, Aaron C. Petrey^2,3^, Kimberly A. Queisser^2,3^, John L. Rustad^2^, James M. Fulcher^5^, Judah L. Evangelista^5^, Michael S. Kay^5^, Joshua D. Schiffman^6,7^, Robert A. Campbell^2,8^, and Christian C. Yost^1,2^*

Furthermore, in the published article, there was an error. In the **Author Contributions** section, the contributions of James M. Fulcher were not mentioned.

A correction has been made in the **Author Contributions** section, third sentence. This sentence previously stated:

“JE and MK developed and performed the NET-Inhibitory Peptide quantitative assay.”

The corrected sentence appears below:

“JF, JE, and MK developed and performed the NET-Inhibitory Peptide quantitative assay.”

The authors apologize for these errors and state that they do not change the scientific conclusions of the article in any way. The original article has been updated.

