# Supplementary material for: Neonatal NET-Inhibitory Factor improves survival in the cecal ligation and puncture model of polymicrobial sepsis by inhibiting neutrophil extracellular traps
Source: Front Immunol. 2023 Jan 17;13:1046574. doi: 10.3389/fimmu.2022.1046574 (PMC9888311; doi:10.3389/fimmu.2022.1046574)
Supplement: Supplementary file 1 [file DataSheet_1.pdf]

Neonatal NET-Inhibitory Factor (nNIF) improves survival in the cecal ligation and puncture model of polymicrobial sepsis by inhibiting neutrophil extracellular traps

Claudia V. de Araujo[1,2], Frederik Denorme[2], W. Zac Stephens[5], Qing Li[7], Mark J. Cody[1,2], Jacob L. Crandell[2], Aaron C. Petrey[2,5], Kimberly A. Queisser[2,5], John L. Rustad[2], Judah Evangelista[4], Michael S. Kay[4], Joshua D. Schiffman[6,8], Robert A. Campbell[2,3], Christian C. Yost*[1,2].

Supplemental Methods:

Pharmacodynamic studies

For the pharmacodynamics studies mice were anesthetized with isoflurane and pre-treated intravenously with nNIF or nNIF-SCR (2 mg/kg in 0.003% DMSO/H_2O). Then, mice were challenged with LPS (19 mg/kg, IP) intraperitoneally on different timepoints: 6, 8, or 12 hours. Survival was followed for 5 days. In a separate experiment, mice were pre-treated with nNIF and SCR peptides and injected with LPS as described above. Sixteen hours after LPS injection, peritoneal fluid and plasma were collected for MPO-DNA complex formation measurements.

Pharmacokinetic studies

PK plasma sample generation – Mature Swiss-Webster mice were injected with nNIF (10 mg/kg, 0.003% DMSO in PBS) via retro-orbital venous injection. Blood samples were obtained via cheek bleed and cardiac puncture at 5, 15, 30, and 45 minutes, and at 1, 4, 8, 12, 24, and 48 hours after nNIF injection. Only three blood samples were drawn from each individual mouse, and multiple mice were used to generate 2-3 plasma samples per timepoint for PK analysis. Blood samples were collected in heparin coated capillary tubes, centrifuged at 1000 rpm for 10 minutes, and snap-frozen in liquid nitrogen. Plasma samples were kept frozen at -80°C until analysis.

Peptide synthesis – Internal standard nNIF-H was synthesized with an isotopically labeled amino acid [Phe-$^{13}C_9{}^{15}N$] at position 2, increasing the molecular weight by 10.0 Daltons. Peptides were synthesized on Gyros Protein Technologies *Prelude X* instrument using standard Fmoc chemistry

at room temperature. Peptides were prepared on 2-chlorotrityl chloride resin (ChemPep, 0.2 mmol/g) to generate C-terminal carboxylic acids. Each AA cycle consisted of deprotection with 20% piperidine in N,N'-dimethylformamide (DMF) (2 x 2 mins), followed by coupling with [1.3/1.3/1] (v/v/v) of [200 mM Fmoc-AA in N-methyl-2-pyrrolidone/195 mM HATU in DMF/600 mM N-methylmorpholine in DMF] (25 mins). Peptide resins were cleaved for 3 hrs with 4 mL [95/2.5/2.5] of [trifluoroacetic acid (TFA)/water/triisopropylsilane], precipitated and washed thoroughly with ether, then dried for several hours. Crude peptides were purified by RP-HPLC using 0.1% TFA in water as the mobile phase and 0.1% TFA in acetonitrile as the eluent, then lyophilized.

Mouse plasma PK quantitative bioanalysis – Flash-frozen mouse samples were thawed on ice and spiked with 100 nM nNIF-H; precipitated with five volumes of 90% acetonitrile, 1% acetic acid, and 0.1% formic acid in water; and filtered through a Phree Phospholipid Removal Extraction Plate (Phenomenex). Standards with known concentrations of nNIF (100 pM to 25 μM) and nNIF-H (100 nM) spiked into mouse plasma were prepared using the same methods. Supernatants were analyzed by LC/MS/MS on an ExionLC UHPLC system coupled to a QTRAP 6500 triple-quad mass spectrometer (AB Sciex) using MRM methods. Following centrifugation, 5 μL of supernatant were injected onto an AdvanceBio Peptide Mapping HPLC column (Agilent) and separated with a gradient of 5-40% acetonitrile in water with 0.1% formic acid at a flow rate of 0.75 mL/min and column temperature of 40°C. Ions were formed by electrospray in positive-ion mode. Mass transitions (m/z) used for quantitation were 846.9/976.1 and 846.9/1014.1 for nNIF, 849.3/979.0 and 849.3/1017.2 for nNIF-H. Plasma concentrations were determined from the nNIF/nNIF-H peak area ratio in extracted-ion chromatograms compared against a standard calibration curve with samples of known nNIF concentration from 0.1 nM to

25 μM. Calibration curve was linear in the 6 nM – 25 μM range (R^2=0.998). Average background signal from blank plasma was subtracted from each sample, and 3 nM (after blank subtraction) was determined as the limit of detection, i.e., the highest background signal found in any blank.

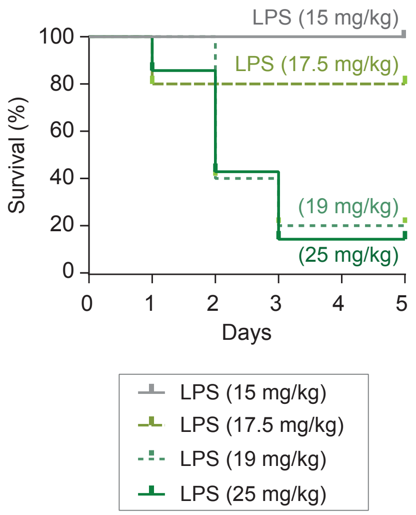

Supplemental Figure 1: ***Intraperitoneal LPS injection leads to mortality in Swiss-webster mice.*** In pharmacodynamic studies, we performed a concentration curve of LPS – 15 (gray solid), 17.5 (lime dashed), 19 (green dashed), and 25 (green solid) mg/kg/dose – and determined percent survival (y-axis) for each LPS concentration. N = 6 separate mice per treatment group. The survival was recorded for 5 days. We used the Log-rank (Mantel-Cox) statistical tool to compare treatment groups.
*denotes P> 0.05**, denotes P<0.01.

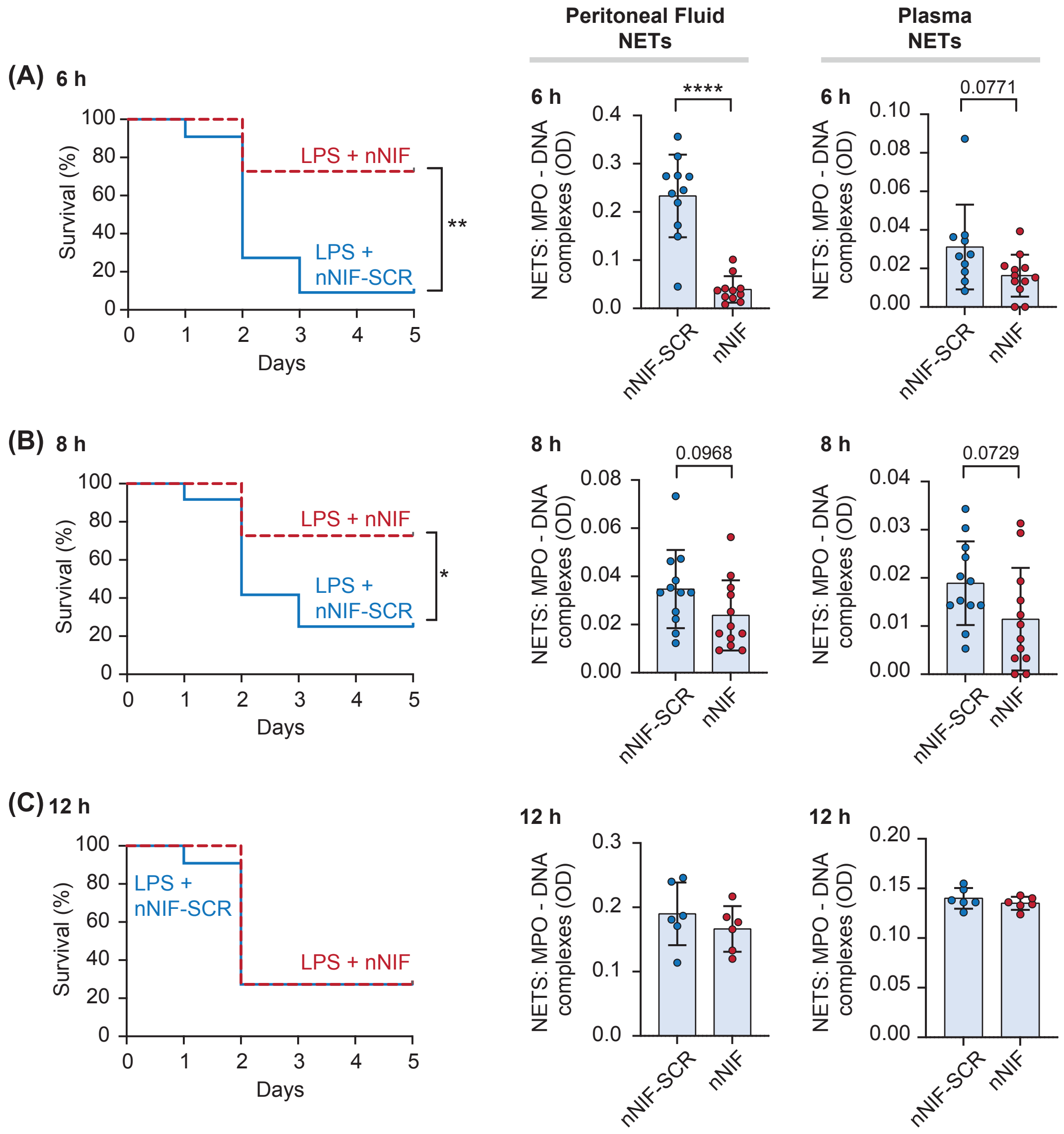

Supplemental Figure 2: ***nNIF treatment protects against mortality in Swiss-webster mice when given up to 8 hours prior LPS injection.*** **(A-C)** In pharmacodynamic studies, we assessed survival and quantified peritoneal fluid and plasma NET formation using the MPO-DNA ELISA to determine levels of MPO-DNA complexes as a surrogate for NET formation. We used a model of LPS-induced peritonitis (19 mg/kg/dose) ± nNIF (2 mg/kg), nNIF-SCR (2 mg/kg) dosed **(A)** 6, **(B)** 8, or **(C)** 12 hours prior to LPS injection. Survival rate was followed over 5 days. Percent survival is shown on the y-axis for mice injected with LPS (19 mg/kg/dose, IP) + nNIF (red dashed), nNIF-SCR (blue solid). N=6 mice per group. Peritoneal fluid and plasma samples were collected 16 hours after LPS injection. N = 10-12 separate mice per group for MPO-DNA complex ELISA. For survival experiment, we used the Log-rank (Mantel-Cox) statistical tool to compare treatment groups. For the MPO-DNA ELISA, we employed the student's t-test statistical tool.
*denotes P< 0.05; ** denotes P<0.01; **** denotes P<0.0001.

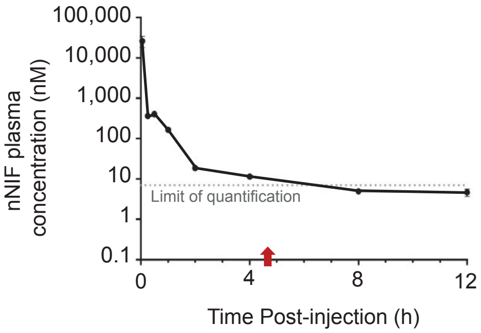

Supplemental Figure 3: ***nNIF pharmacokinetics in Swiss-webster mice.*** Concentration of nNIF in plasma of Swiss-webster mice after a single dose (10 mg/kg), measured at indicated time points after dosing (N = 2-3 mice/time point). Background signal from control plasma was subtracted from all points; dashed line indicates the assay limit of detection (3 nM). Timepoints > 8 hours were below the limit of detection.

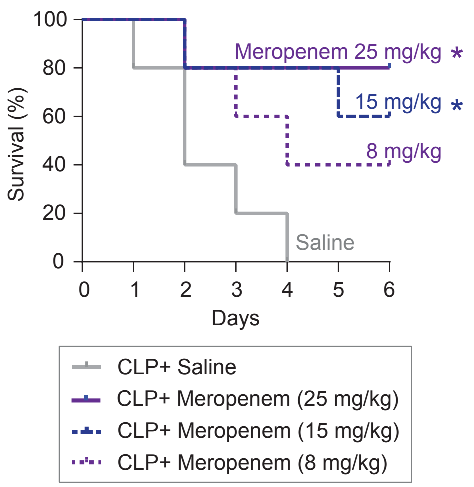

Supplemental Figure 4: ***Meropenem dosage correlates with survival in CLP-treated Swiss-webster mice.*** We assessed survival over 6 days in CLP-treated mice receiving varying doses of meropenem at 4 hours and 10 hours after CLP – 8 (purple dashed), 15 (blue dashed), or 25 (purple solid) mg/kg per dose – or normal saline vehicle (gray solid). N = 5 separate mice per group compared to vehicle control. Sham mice (N = 3) were subjected to sham surgery. We used the Log-rank (Mantel-Cox) statistical tool. * denotes $P<0.05$.
